# Engineering antibody-armed oncolytic viruses: design strategies, synergistic mechanisms, and clinical translation

**DOI:** 10.3389/fimmu.2026.1805770

**Published:** 2026-04-07

**Authors:** Xiaozhen Kang, Xue Yang, Hui Wu, Xiangmin Tong, Shibing Wang

**Affiliations:** 1 Zhejiang Key Laboratory of Zero Magnetic Medicine, Affiliated Hangzhou First People’s Hospital, School of Medicine, Westlake University, Hangzhou, Zhejiang, China; 2 School of Pharmaceutical Sciences, Zhejiang Chinese Medical University, Hangzhou, Zhejiang, China; 3 Zhejiang Provincial People’s Hospital (Affiliated People’s Hospital), Hangzhou Medical College, Hangzhou, Zhejiang, China; 4 Department of Clinical Laboratory, Affiliated Hangzhou First People’s Hospital, School of Medicine, Westlake University, Hangzhou, Zhejiang, China

**Keywords:** antibody delivery, combination (combined) therapy, oncolytic viruses, therapeutic antibodies, tumor micro environment (TME)

## Abstract

Therapeutic antibodies are widely used in cancer biotherapy due to their target specificity, mediating tumor cell inhibition, angiogenesis suppression, and immune modulation. However, systemic administration often leads to off-target effects, as many antibody targets are also expressed in normal tissues, limiting intratumoral drug concentration and causing adverse events. Oncolytic viruses (OVs), which selectively infect and lyse tumor cells while activating host anti-tumor immunity, offer a promising platform for localized antibody delivery. Their inherent tumor tropism, intratumoral administration, and high genetic manipulability enable the engineering of OVs to express exogenous antibodies within the tumor microenvironment, enhancing therapeutic specificity and synergizing oncolytic and immune-mediated effects. In this review, we summarize the biological properties of OVs, strategies for engineering antibody payloads, the mechanistic interplay between OV-induced oncolysis and immune modulation, and current challenges and opportunities for clinical translation. By integrating these aspects, we provide insights into optimizing OV-based antibody therapies for enhanced tumor-targeted efficacy and reduced systemic toxicity.

## 1 Introduction

Cancer biotherapies have been widely applied in clinical oncology, with therapeutic antibodies representing the most extensively used modality. These antibodies exhibit target specificity, primarily functioning to inhibit tumor cell proliferation, suppress tumor angiogenesis, and modulate immune responses (1, 2). However, although the corresponding targets may be significantly upregulated within the tumor, they are often still present in peripheral tissues. Consequently, systemically administered therapeutic antibodies frequently induce off-target effects, which not only reduce the effective concentration of the drug within the tumor microenvironment but may also result in severe adverse events (3, 4). Therefore, optimizing tumor-localized delivery while avoiding systemic toxicity remains a central conceptual challenge in the field.

Oncolytic viruses are a class of viruses capable of selectively infecting and lysing tumor cells while simultaneously activating host anti-tumor immune responses (5, 6). Compared with conventional chemotherapy or radiotherapy, OVs possess inherent tumor tropism, enabling direct tumor cell killing through viral replication (7–9). Moreover, current OV administration primarily relies on intratumoral injection, and most OVs do not integrate into the host genome, This, together with their high genetic manipulability, allows them to be engineered to carry exogenous genes such as cytokines, small RNAs, or antibodies (10–12).

Crucially, OVs offer a distinct advantage over the conventional combination of unmodified OVs with systemically administered antibodies. By utilizing OVs as local “bio-factories,” therapeutic antibodies can achieve highly concentrated, continuous expression strictly localized within the TME (12). This restricts systemic exposure and minimizes immune-related adverse events. Recent studies have demonstrated that integrating antibodies into OV genomes can simultaneously exert direct oncolytic and immune-regulatory effects, synergistically enhancing anti-tumor activity (13, 14). In this review, we provide a comprehensive overview of engineering antibody-armed OVs, highlighting their biological properties, the rationale for selecting antibody formats, mechanistic synergy, and future clinical directions.

## 2 Biological basis of oncolytic viruses as delivery platforms

Oncolytic viruses are capable of selectively infecting and lysing tumor cells while simultaneously activating host immune responses. Compared with conventional therapies, OVs exhibit higher tumor specificity and can modulate the tumor microenvironment to enhance anti-tumor immunity (15, 16). Their intrinsic mechanisms of action include: ① efficient viral replication within tumor cells leading to direct oncolysis; and ② OVs-induced lysis of tumor cells leads to the release of TAAs, DAMPs, and PAMPs, which activate antigen-presenting cells and the innate immune system, eliciting robust antitumor immune responses.

Commonly studied and clinically applied OVs include adenovirus (Ad), herpes simplex virus (HSV), measles virus (MV), Newcastle disease virus (NDV), and vaccinia virus (VV) (17). OV design strategies primarily focus on tumor-specific replication and enhanced infectivity. For instance, in oncolytic adenoviruses, tumor-specific promoters (TSPs) can regulate viral gene expression, deletion or mutation of E1A/E1B genes can increase tumor selectivity, and capsid modifications can improve infection efficiency in tumor cells. Representative examples include AdΔ24 (E1A mutant) and Oncorine (H101, E1B-deleted), which selectively replicate in tumor cells with defects in the pRb or p53 pathways, respectively (18–20); Ad5/3-D24 employs capsid modifications to enhance infection of tumors with low CAR expression (21). Additionally, OVs can carry transgenes such as GM-CSF to further enhance antigen presentation and T cell activation. Clinically, T-VEC (HSV expressing GM-CSF), Oncorine (H101), DNX-2401, CG0070, and ONCOS-102 have demonstrated promising anti-tumor potential across multiple malignancies, including melanoma, head and neck cancer, glioblastoma, and ovarian cancer (22–25).

Beyond direct oncolysis, OVs can remodel the tumor immune microenvironment. Viral infection induces immunogenic cell death (ICD), releasing cytokines and danger-associated molecules such as ATP, HMGB1, and exposed calreticulin, which promote dendritic cell maturation and antigen presentation (10, 26, 27). Such modulation not only amplifies the intrinsic anti-tumor efficacy of the virus but also provides a necessary inflammatory foundation for combination immunotherapies, making OVs an ideal platform for delivering customized antibody payloads.

## 3 Strategies for engineering therapeutic antibody payloads

**Figure 1:**
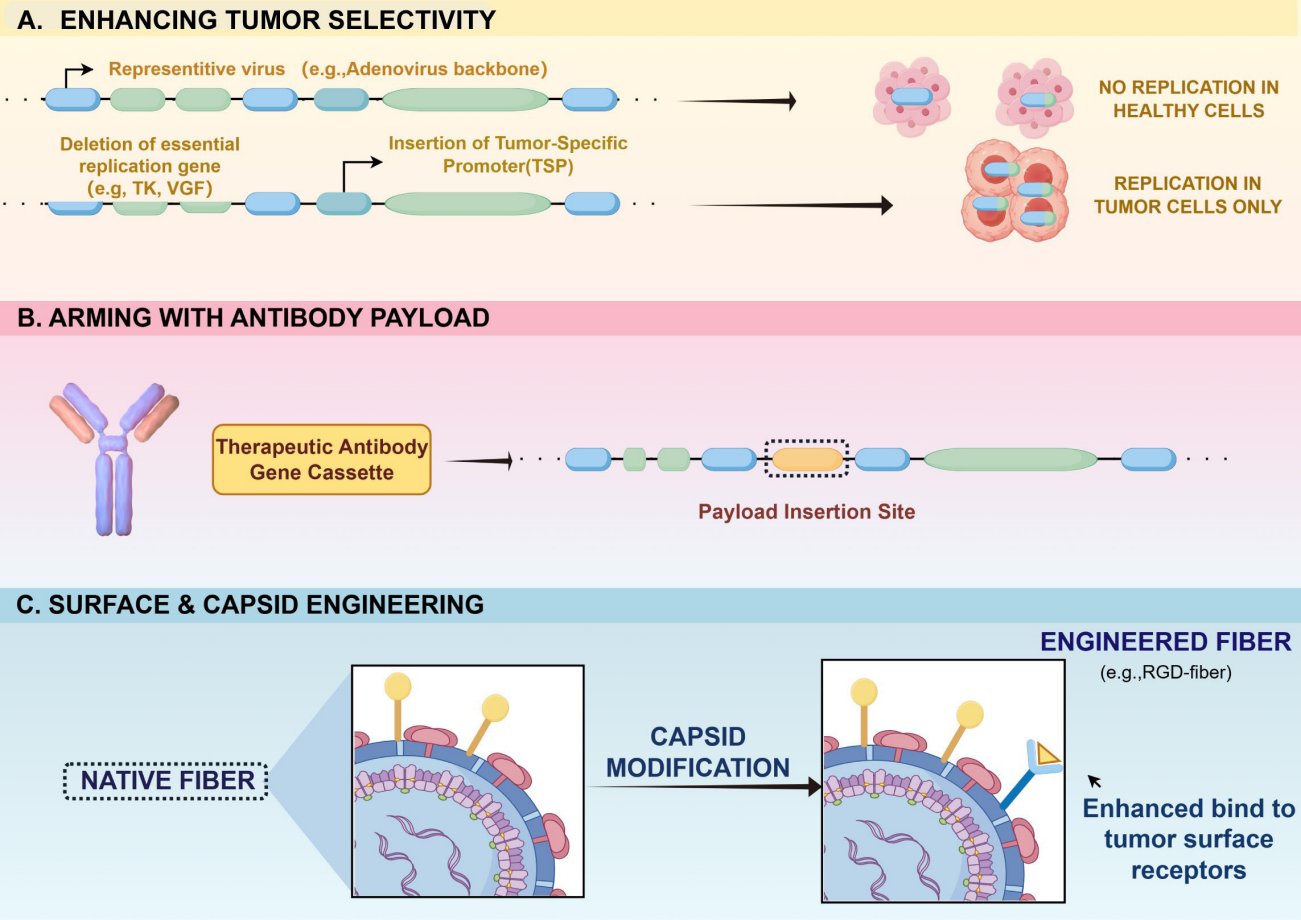
Rational engineering of the oncolytic virus platform. This figure details the molecular strategies used to modify the viral vector itself before delivery. **(A)** Specificity is enhanced through gene deletions or the insertion of TSP to restrict viral replication to malignant cells. **(B)** The viral genome is armed with a specific therapeutic antibody gene cassette. **(C)** Viral capsid or fiber proteins are engineered to modify surface tropism and enhance infectivity in dense solid tumors.

One of the major advantages of OVs is their ability to carry and locally express antibodies within the TME. The rational design of these engineered OVs requires matching the specific viral vector’s packaging capacity with the appropriate antibody format (**Figure 1**).

Rationale for Antibody Format Selection: Large DNA viruses, such as Vaccinia virus and HSV, possess massive genomic capacities, allowing for the stable integration of full-length IgGs (28, 29). Full-length IgGs offer Fc-mediated effector functions (such as ADCC or ADCP) and prolonged half-lives in the TME (30). Conversely, vectors with limited packaging capacity (e.g., specific Adenoviral vectors) frequently utilize single-chain variable fragments (scFvs) or Nanobodies (31, 32). These smaller formats ensure high expression yields, superior tissue penetration within dense solid tumors, and provide modular building blocks for designing bispecific T-cell engagers (BiTEs) that require tight immunological synapses (33).

Immune Checkpoint Antibodies: OVs have been extensively engineered to express immune checkpoint–blocking antibodies, combining direct tumor cell lysis with TME remodeling to overcome immunosuppressive signals (34, 35). For PD−1/PD−L1, recombinant human PD−1 antibody–armed herpes simplex virus 1 (rHSV−1−APD1) incorporates a PD−1–specific scFv into the viral genome. This enables local PD−1 blockade in the TME, alleviating immunosuppression and achieving a dual synergistic effect of oncolysis and checkpoint inhibition (36). For CTLA−4, BT−001 is an oncolytic vaccinia virus engineered to express a humanized, regulatory T cells (Treg) -depleting anti-CTLA-4 antibody alongside GM−CSF. BT−001 persists and replicates within tumors, with antibody expression confined locally, avoiding systemic exposure. Early clinical observations have reported tumor shrinkage in some patients, demonstrating improved safety and antitumor efficacy (14). Additionally, emerging checkpoint targets such as TIGIT have been integrated into OVs (37, 38). Vaccinia viruses expressing TIGIT-specific scFvs (VV−scFv−TIGIT) enhance CD8^+^ T cell infiltration and activation in multiple murine tumor models, and can act synergistically with PD−1 or LAG−3 blockade to increase complete response rates (37). 

Antibodies Targeting Immunosuppressive Cells: OVs have also been engineered to target immunosuppressive cells in the TME, with CD47 and TNFR2 as representative targets. CD47 antibodies block the “don’t eat me” signal, promoting macrophage-mediated phagocytosis of tumor cells. Systemic administration of CD47 antibodies carries hematologic toxicity; however, intratumoral delivery via OVs confines antibody activity to the TME, remodeling macrophage function locally while avoiding systemic adverse effects (39, 40). TNFR2-targeting antibodies primarily act on Tregs and MDSCs, and in preclinical studies, TNFR2-armed oncolytic adenoviruses reduced Treg and MDSC populations in pancreatic and colon cancer models, significantly enhancing antitumor efficacy (12, 13).

Multi-Target Antibodies: Multispecific antibodies simultaneously targeting two or more molecules can further potentiate antitumor immune responses by bridging T cells with tumor cells, enhancing cytotoxicity. BiTE- or TriTE-armed OVs have been widely explored (41–44). For example, Yu et al. designed an oncolytic vaccinia virus carrying a T cell engager specific for EphA2 (EphA2-TEA-VV), which selectively activated T cells and inhibited tumor growth in murine models (45). Similarly, an oncolytic adenovirus armed with a MUC16-targeting BiTE redirected infiltrating CTLs to tumor cells, enhancing antigen-specific killing and bystander effects. In murine models, the combination of oncolytic Ad and MUC16-BiTE achieved synergistic and cumulative antitumor effects (46). In our own study, we developed an OV expressing a dual-target antibody against CD47 and TNFR2. This virus not only suppressed Tregs and MDSCs in the TME, but also promoted macrophage phagocytosis, thereby further activating T cells and enhancing antitumor efficacy in murine colon and pancreatic cancer models (13).

## 4 Synergistic mechanisms between viral oncolysis and antibody-mediated immunomodulation

**Figure 2:**
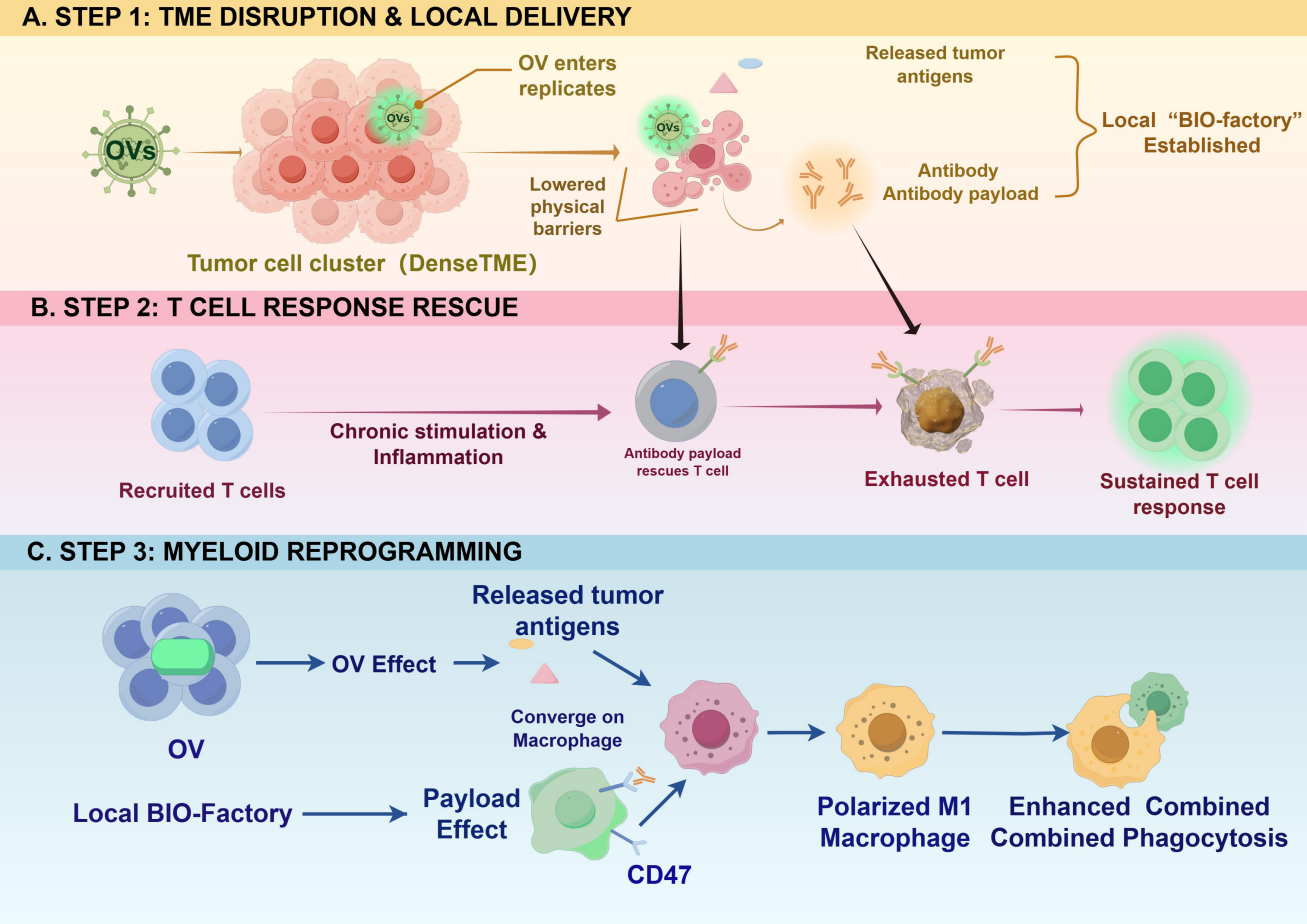
Simplified chronological synergistic immunological effects of antibody-armed OVs following chronology. This diagram illustrates the sequential synergistic interactions between oncolytic virotherapy and localized antibody delivery, simplified into chronological stages. **(A)** Step 1: TME Disruption and Local Delivery. Simple OVs infect and lyse simplified tumor cells, breaking down physical barriers and establishing a ‘Local Bio-factory’ that facilitates high concentrated secretion of therapeutic antibody payloads (e.g., anti-PD-L1). **(B)** Step 2: T Cell Response Rescue. Chronic antigen stimulation and viral inflammation inevitably cause T cells to enter an exhausted state. Localized payload secretion intervenes, binding to the exhausted T cell to “rescue” it, leading to a sustained T cell response. **(C)** Step 3: Myeloid Reprogramming. Parallel effects from OV replication (releasing tumor antigens) and localized payload secretion (CD47 blocking) converge on a single macrophage. This convergence polarizes the macrophage to a simplified ‘M1-like’ state, enabling enhanced combined phagocytosis that would not occur with either single therapy.

The therapeutic superiority of antibody-armed OVs over the simple systemic administration of antibodies arises from a multi-dimensional spatial and temporal synergistic interplay. Rather than functioning as parallel therapies, the virus and the encoded antibody act in a highly coordinated, sequential manner to overcome distinct barriers in the TME (**Figure 2**).

Overcoming Physical Barriers for Enhanced Delivery: Solid tumors are characterized by high interstitial fluid pressure and a dense extracellular matrix, which severely impede the penetration of systemically delivered large-molecule antibodies (47). OV-induced lysis functions as an “immunological primer” and physical disruptor. By lysing tumor cells, OVs lower physical barriers and create a localized “bio-factory” effect (5). This ensures that therapeutic antibodies are produced *in situ*, achieving concentrations directly at the target site that would be highly toxic if administered systemically.

Temporal Synergy: Sustaining the ICD-Induced T Cell Response: While viral infection triggers acute ICD and provides a rich source of antigens for T cell activation, this robust pro-inflammatory surge predictably leads to a compensatory feedback loop. Chronic antigen stimulation and viral inflammation inevitably upregulate exhaustion markers (e.g., PD-1, TIM-3) on infiltrating T cells (48). Here, the armed virus provides critical temporal synergy: exactly as the T cells begin to undergo exhaustion in response to the viral challenge, the locally secreted checkpoint inhibitors (e.g., anti-PD-1 scFv) immediately intervene. The antibody payload “rescues” the very T cells that the virus recruited, ensuring that even exhausted T cells can maintain their critical anti-tumor efficacy, thereby preventing the premature termination of the immune response (49).

Spatial Reprogramming of the Myeloid Landscape: OVs and specific antibody payloads collaboratively remodel the immunosuppressive myeloid compartment. For instance, OV infection induces robust IFN-I/γ production, which promotes an inflammatory milieu. When combined with locally expressed CD47-blocking antibodies, this creates a “dual-hit” synergy: the antibody removes the inhibitory SIRPα-mediated signal, while the viral-induced cytokines actively provide the “eat me” signal required to unleash robust macrophage phagocytosis (50, 51). This mechanism successfully reverses the hostile myeloid environment that frequently limits the efficacy of conventional virotherapy.

## 5 Barriers to clinical translation and future directions

Although antibody-armed OVs have demonstrated significant therapeutic potential, their clinical application remains in the early phases (**Table 1**). Several barriers hinder their broad translation. Safety and tolerability require careful optimization, as OVs may trigger systemic inflammatory responses (52). *In vivo* viral spread is often restricted by host neutralizing antibodies, tissue barriers, and extracellular matrices, limiting delivery efficiency (53). Furthermore, clinical quality control for these complex, dual-action biologicals requires more rigorous potency assays than conventional monoclonal antibodies.

**Table 1:** Summary of antibody-armed oncolytic viruses in clinical stage.

Drug name	Virus type	Antibody payload	Route of admin.	Combination therapy	Cancer indications	Clinical stage	Registry ID
BT-001	Vaccinia	Anti-CTLA-4 (Full IgG)	IT	Monotherapy/w/Pembrolizumab	Advanced solid tumors	Phase I/IIa	NCT04725331
VT1093	HSV-1	Anti-PD-1 (scFv)	IT	Monotherapy	R/M HNSCC	Phase I	NCT05710211
ONCR-177	HSV-1	Anti-CTLA-4/PD-1 (Heavy chain) + IL-12/CCL4	IT	Monotherapy/w/Pembrolizumab	Advanced solid tumors	Phase I	NCT04348916
VG161	HSV-1	Anti-PD-1 + IL-12/IL-15	IT	Monotherapy	Advanced solid tumors	Phase I	NCT04533035
RP2	HSV-1	Anti-CTLA-4 (GALV-GP)	IT	Monotherapy/w/Nivolumab	Advanced solid tumors	Phase I	NCT03767348
T3011	Vaccinia	Anti-PD-L1/IL-12	IV/IT	Monotherapy/w/Pembrolizumab	Advanced solid tumors	Phase I	NCT04301544
rHSV-1-APD1	HSV-1	Anti-PD-1 (scFv)	IT	Monotherapy	Advanced HCC	Phase I	ChiCTR2500098652

Future directions must focus on optimizing viral shielding (e.g., nanoparticle coatings to evade neutralizing antibodies for IV delivery), refining synthetic promoters to restrict antibody expression strictly to the tumor site, and exploring rational combinations with adoptive cell therapies (like CAR-T) to fully leverage the inflamed TME (54, 55).
